# The Role of Symptom Appraisals on Quality of Life in Couples Diagnosed with Cancer

**DOI:** 10.1093/geroni/igaf122.2940

**Published:** 2025-12-31

**Authors:** Silvia Cilluffo, Karen Lyons

**Affiliations:** University of Milan, Milan, Lombardia, Italy; Boston College, Chestnut Hill, Massachusetts, United States

## Abstract

Although survival rates for many cancers are increasing, most cancer survivors continue to experience symptoms that can impact their quality of life and that of their spouse/partner. Guided by the Theory of Dyadic Illness Management, the current study focused on symptoms shared appraisal in survivor- spouse’s dyads affects their quality of life. The sample was composed of 49 young-midlife couples within two years of a cancer diagnosis living in urban and rural areas. Actor partner interdependence models examined the association of survivor’s symptoms (reported by both the patient and their spouse) on the physical and mental quality of life (QoL) of couples. Significant actor effects were found for survivor’s reports of symptoms.; Higher survivor fatigue, pain severity, and pain interference on activities of daily living were significantly associated with worse survivor physical QoL(p = <.001); higher survivor fatigue and pain severity were significantly associated with worse survivor mental QoL (p<.05)., For spouses, a significant actor effect was found for spouse mental QoL; when spouse’s perceived survivor pain to be high they reported worse mental QoL (p<.05). A significant partner effect was found for pain interference. When survivors rated their pain interference high, their spouses were significantly more likely to report poor physical QoL (p<.001). Discussion will focus on the importance of examining appraisals of cancer symptoms within dyads to optimize QoL of both members. Discussion will also focus on the need for further research on the role of symptom appraisal on health behaviors and symptom management in couples living with cancer.

